# An Overview of Some Nonpiezoelectric Properties of BaTiO_3_ Ceramics Doped by Eu Ions

**DOI:** 10.3390/ma15155363

**Published:** 2022-08-04

**Authors:** Magdalena Krupska-Klimczak, Przemyslaw Gwizd, Irena Jankowska-Sumara, Dorota Sitko, Piotr Jeleń

**Affiliations:** 1 Institute of Physics, Pedagogical University of Krakow, ul. Podchorazych 2, 30-084 Cracow, Poland; 2 Faculty of Materials Sciences & Ceramics, AGH University of Science & Technology, al. A. Mickiewicza 30, 30-059 Cracow, Poland

**Keywords:** BaTiO_3_, ferroelectric properties, electrocaloric effect

## Abstract

Ferroelectric ceramics BaTiO_3_:x%Eu (x = 0, 0.1, 1, 2, 3) were synthesized by a conventional method. Structural investigation confirmed that all ceramics possessed tetragonal (*P4mm*) symmetries at room temperature for the undoped ceramics as well as for the doped ceramics. Furthermore, a slight downshifting of the Curie temperature (*T*_C_) with an increasing Eu^3+^ doping amount has been noted. The Raman spectra unveiled the existence of new modes for higher-doped BaTiO_3_:x%Eu (BTEx) which are related to local disorders and defects. The ferroelectric properties were found to depend on both doping and the microstructure. The electrocaloric effect was also studied for those ceramics. It was observed that Δ*T* decreases with doping; however, the temperature range of its occurrence widens considerably.

## 1. Introduction

Ferroelectric titanates, such as PbTiO_3_, SrTiO_3_, and BaTiO_3_ (BTO), with typical perovskite ABO_3_ structures have been widely studied for many years due to their outstanding dielectric properties, ferroelectric properties, and electro-optic and electromechanical properties [1,2,3,4]. The BaTiO_3_ (BTO) based ferroelectric ceramics exhibit a number of superior properties, such as large spontaneous polarization, high dielectric permittivity in the ferroelectric phase, and relatively good piezoelectric response. In addition, BTO is a common ferroelectric material that undergoes successive phase transitions at different temperatures. The reason for the still increasing attraction of BTO is not only the use of its properties for industrial application [5], but also its ability to change its properties relatively easily by introducing various kinds of dopants [6,7]. Due to the serious environmental problems that have arisen from the long-term use of toxic heavy metal lead, much attention has recently been given to the use of lead-free materials. Rare earth-doped BTO attracts scientific attention because of its interesting electro-optical properties, such as luminescence. Since the ionic radius of Eu^3+^ is intermediate between the Ba^2+^ ion and Ti^4+^, it appears that the Eu^3+^ ion can occupy both A- and/or B-positions depending on the Ba/Ti ratio [8,9,10,11]. It is also known that controlled substitutions in parent compounds with iso or heterovalent ions is one way to better understand the nature of PTs in ferroelectrics. For example, it was found that the position of the dopant site plays an important role in the electrical properties of BTO doped by Eu^3+^ ions. In our earlier publications, we have found that a small amount of Eu acts as a donor dopant, i.e., it predominantly occupies the Ba^2+^- site [10,11,12]. It has been shown that doping with Eu ions lowers both the ferroelectric phase transition temperature and the value of electrical permittivity at *T*_C_. When the amount of doping exceeds 3% Eu in BTO, the ε(T) characteristic becomes strongly flat and diffuse. In the paper by Patel [13], it was found that, above 3% Eu in BTO, the contribution of the hexagonal phase in BTE ceramics is possible. Taking this into account, we have investigated the effect of doping BaTiO_3_ with heterovalent Eu^3+^ ions up to an amount of 3% on some electrical properties of BaTiO_3_.

We deeply believe that it is very important to gain knowledge about the basic properties of this type of material. Therefore, in this work we take a closer look at some nonpiezoelectric properties including the very peculiar phenomenon of an electrocaloric effect of BaTiO_3_ ceramics doped by Eu ions. Some preliminary measurements of the electrocaloric effect were made earlier for pure BaTiO_3_ doped with 2% of Eu. The results of these measurements are included in the paper by Gwizd et al. [14]. The results showed the intriguing phenomenon of negative electrocaloric effect (ECE) occurring just before the phase transition. For this reason, we decided to repeat and complete these measurements for a larger number of samples of different concentrations of Eu. Hence, here we present systematic measurements for a set of samples between 0.1 and 3% Eu. In this way, we want to show how doping influences the different physical properties of ceramics doped with Eu ions.

## 2. Materials and Methods

A set of BaTiO_3_:x%Eu (with x = 0, 0.1, 1, 2, and 3, hereafter in publication denoted as BTEx or BTE0.1, BTE1, BTE2, and BTE3, respectively, and BTO for the pure BaTiO_3_) ceramics were synthesized by the so-called solid-state sintering method. Analytically pure barium carbonate BaCO_3_ (99.98% Sigma Aldrich, Darmstadt, Germany), titanium oxide (IV) TiO_2_ (99.5% Sigma Aldrich), and europium oxide Eu_2_O_3_ (III) (99.9% Sigma Aldrich) were used as starting oxides. Prior to weighing, the powders were dried at 470 K for 4 h to reduce the moisture content and were then kept in desiccators. The mixture of raw materials was initially ground and mixed in an agate ball mill in ethanol for 24 h. The thoroughly dried material was then calcined for about two hours at 1523 K. In the next stage the synthesized BTO was ground again into powder and then doped with europium oxide (Eu_2_O_3_) with amounts of 0.01%, 0.1%, 1%, 2%, and 3% by weight, respectively, and the obtained mixtures were ground again in the ball mill for 24 h and then dried. The prepared powders were pressed in a hydraulic press under 150 MPa pressure to form compact pieces [15]. Approximately 1 g of the powder was used to obtain pellets of 10 mm in diameter and c.a.3 mm in height. The pressure values were selected experimentally. Tested samples were sintered in the air atmosphere and at the optimized temperature for 2 h. The sintering temperature is a key parameter in ceramic technology. Depending on the type of doping compounds, the samples were sintered at temperatures in the range of 1690–1730 K. The sintering temperature was selected experimentally. Obtained ceramics were subjected to mechanical treatment (cutting, polishing) and then held at a temperature of 750 K to eliminate the internal stresses that arose during the sintering and treatment operations.

The structure of the unpoled samples was determined by an X-ray diffractometer. The XRD studies were carried out using a Philips X′Pert Pro MD diffractometer. A standard Bragg–Brentano geometry with Cu Kα radiation was used. Measurements of the structure were carried out in air in the scanning range of 2Θ 20°–100° with a step of 0.017. The microstructure tests and the qualitative and quantitative analyses of the chemical composition of samples have been investigated by using the SEM method (Scanning Electron Microscopy) alongside Energy Dispersive X-ray Spectroscopy (EDS). EDS spectroscopy was used to confirm the chemical composition and homogeneity of the resulting ceramics. The average grain sizes were calculated using the Mean Linear Intercept method [16].

The volume densities of all samples were determined by the Archimedes method. The Raman spectra were obtained using a Bio-Rad FTS 6000 spectrometer equipped with an Nd-Yag laser with a 1064 nm excitation line. The laser power was 200 mW. The spectra were collected with 4 cm^−1^ resolution. A Netzsch DSC F3 Maia scanning calorimeter operating in an argon atmosphere at a flow rate of 40 mL/min was used to measure specific heat in the temperature range from 220 to 500 K. The sample, in the form of a single piece of material with an average weight of 10–20 mg, was placed in an aluminum crucible. A constant rate of 10 K/min was used for measurements (on heating as well as on cooling). For electric measurements, ceramic samples were cut into slices of ~0.5 mm thickness. The plates of the average surface ~3 × 1 mm^2^ were coated with silver electrodes and placed in the computer-controlled furnace. In this furnace, the temperature was controlled by a thermocouple with an accuracy of 0.1 K. All of the measurements have been performed at the temperature rate of 1 K/min. A Precision LCR meter (Agilent E4980A) was used for measurements of relative permittivity at different frequencies, between 20 Hz and 20 kHz, and a temperature range from 150 to 550 K was used. The Sawyer–Tower method in the quasistatic limit was used to measure the ferroelectric hysteresis loop *P*(*E*). The measurements were performed on the heating and cooling process in the temperature range from 300 K to 500 K. The frequency of the test signal was set at 6 Hz, and the amplitude of the electric field was varied from 0 to 6 × 10^5^ V/m.

## 3. Results and Discussion

### 3.1. Structural Characterization

**Figure 1:**
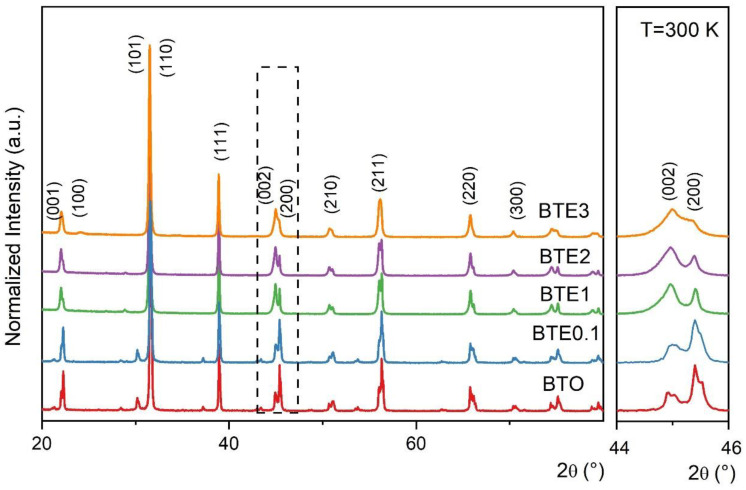
X-ray diffractograms of BaTiO_3_:x%Eu samples: BTO, BTE0.1, BTE1, BTE2, and BTE3. The dashed box is enlarged and placed on the right showing the doublet (200)/(002) in more detail.

Figure 1 shows the X-ray diffractograms obtained for BTEx samples. All samples exhibited pure perovskites without an obvious secondary phase within the apparatus resolution, suggesting that Eu^3+^ has dissolved into the BaTiO_3_ lattice. For x = 0, i.e., pure BTO, we identified the perovskite structure with a tetragonal symmetry (the bottom line, JPCDS 83-1878). This tetragonality is visualized in terms of the 2θ peak near 45°. Namely, the peak corresponding to the (200) plane from the cubic phase splits into a doublet corresponding to the (200) and (002) planes of the tetragonal phase of the BTO. For other samples, the (200)/(002) peaks are found to still be split and asymmetric. For the higher-doped BTE sample (BTE3), this splitting becomes a little blurred, implying that the difference between lattice parameters *a* and *b* corresponding to tetragonal symmetry is decreasing. This could also indicate the weak influence of the low-temperature orthorhombic phase [17,18].

### 3.2. Microstructure

**Figure 2:**
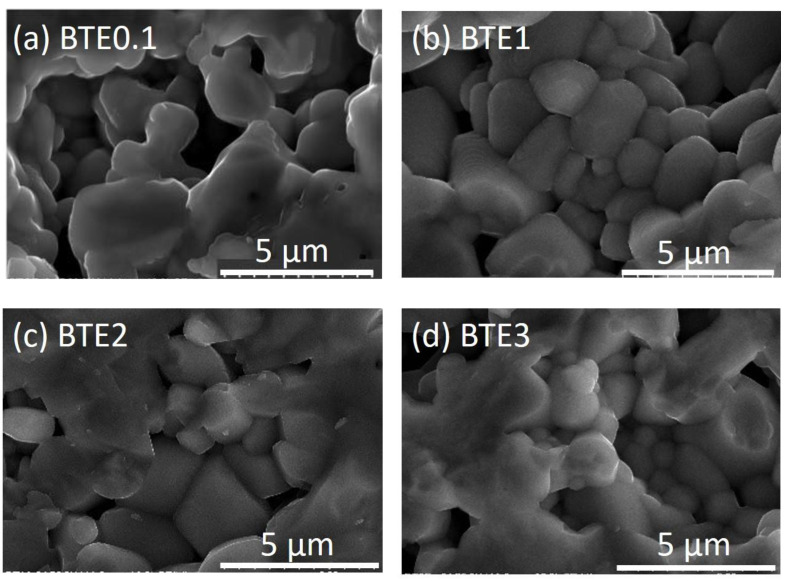
Scanning Electron Microscopy (SEM) images of the microstructure of fractures of (**a**) BTE0.1, (**b**) BTE1, (**c**) BTE2, and (**d**) BTE3.

Figure 2 shows SEM micrographs of BTEx ceramics. The pictures show that the fracture surfaces run both at the grain boundary and at the intragranular boundary. The surface of fractures is composed of separate grains with well-developed boundaries. One can observe that the ceramics are well-sintered with a crispy breakthrough. The effect of Eu^3+^ is clearly visible: the doped ceramics show smaller grains compared to BTO which tend to decrease with increasing dopant content. The BTO ceramics exhibit large grains with an average grain size of about 7.8 mm. The evaluated average grain size derived for Eu-doped samples is as follows: 4 ± 0.5, 2.5 ± 0.6, 1.8 ± 0.5, and 1.6 ± 0.6 μm for BTE0.1, BTE1, BTE2, and BTE3, respectively.

It appears that crystallite sizes are dependent on the doping level and the type of doping compound. After analyzing the images obtained with a microscope, it was concluded that samples containing 3wt.% Eu_2_O_3_ had the smallest grain size. Decreasing grain size is also verified by the blurring of the splitting at the peak of (200) (see Figure 1) since, together with decreasing grains, the difference between the lattice parameters (*a* and *b*) of the tetragonal phase is diminished. One of the reasons why the grain size tends to decrease with the increased content of a doping compound is that doping inhibits grain growth. Apart from that, the results show that crystallites in BTE ceramic materials are well-formed and densely packed. SEM analysis revealed no presence of separate phases, showing good cohesion of thus obtained ceramic material.

As the Eu content increases, the porosity of the ceramics also increases slightly, which results in a decrease in their density. The density values of the doped ceramics are found to be 5.82 ± 0.06, 5.73 ± 0.06, 5.85 ± 0.07, 5.68 ± 0.07, and 5.69 ± 0.06 g/cm^3^ for BTO, BTE0.1, BTE1, BTE2, and BTE3, respectively. The porosity is most often related to technological conditions of ceramics manufacture and stoichiometry problems. The latter is due in large part to the variable electronegativity of the ions forming the compound, which will be discussed later in this work.

**Figure 3:**
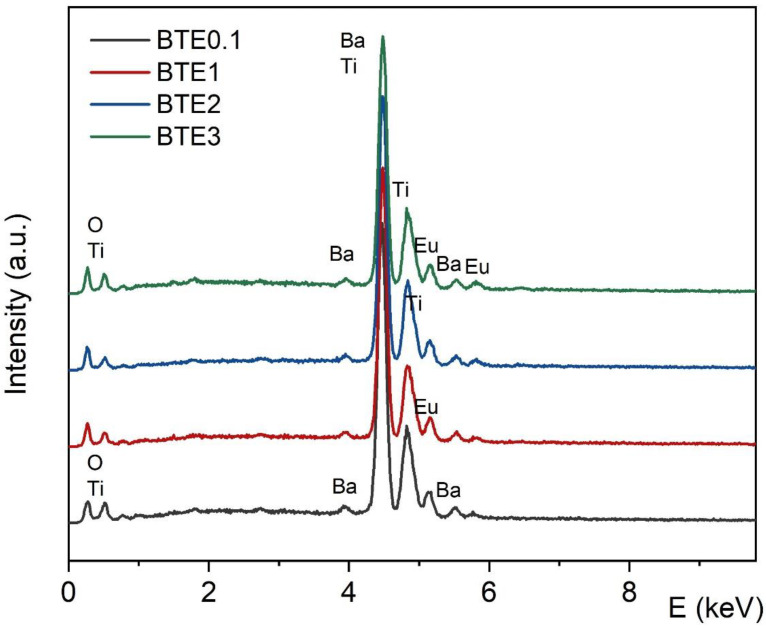
The Energy Dispersive Spectrometry (EDS) analysis of chemical elements of the BTEx ceramic samples: BTE0.1, BTE1, BTE2, and BTE3.

**Figure 4:**
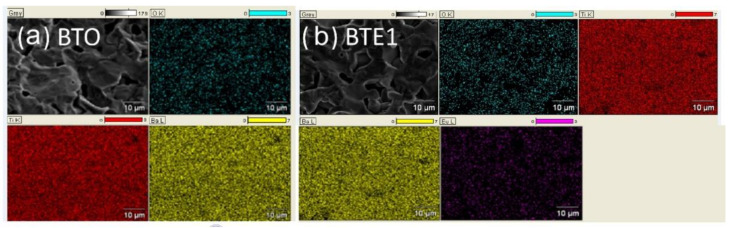
Surface analysis of the elemental composition. EPMA mappings for two selected samples: (**a**) BTO and (**b**) BTE1.

The EDS spectra for BTEx ceramics with the marked spectral positions of the individual elements are displayed in Figure 3. It can be seen that qualitative EDS examination confirms the lack of any traces of contaminants (secondary carbon, etc.) in analyzed samples. Thus, the EDS analysis, together with EPMA mapping, confirmed the good stoichiometry of the ceramics and uniform distribution of the elements (Figure 4).

### 3.3. Dielectric Properties

**Figure 5:**
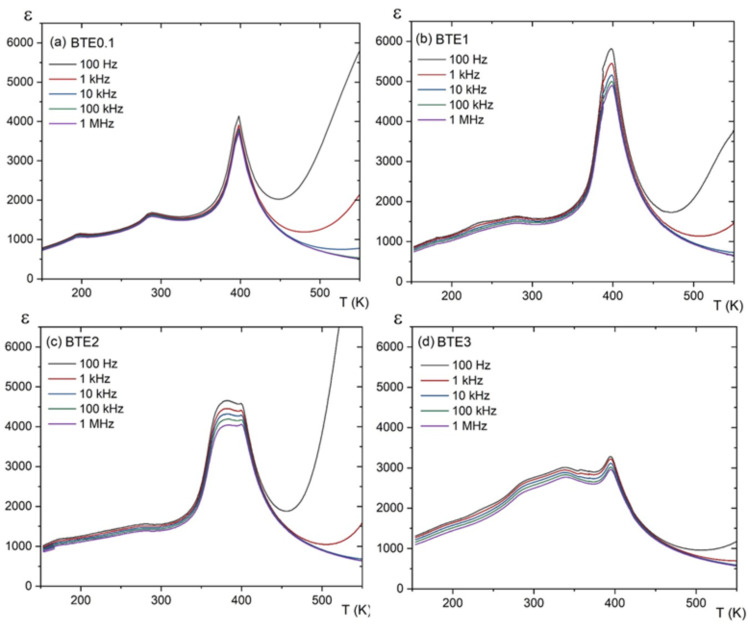
Temperature dependences of the dielectric constant for BTEx ceramics: (**a**) BET0.1, (**b**) BTE1, (**c**) BTE2, and (**d**) BTE3.

**Figure 6:**
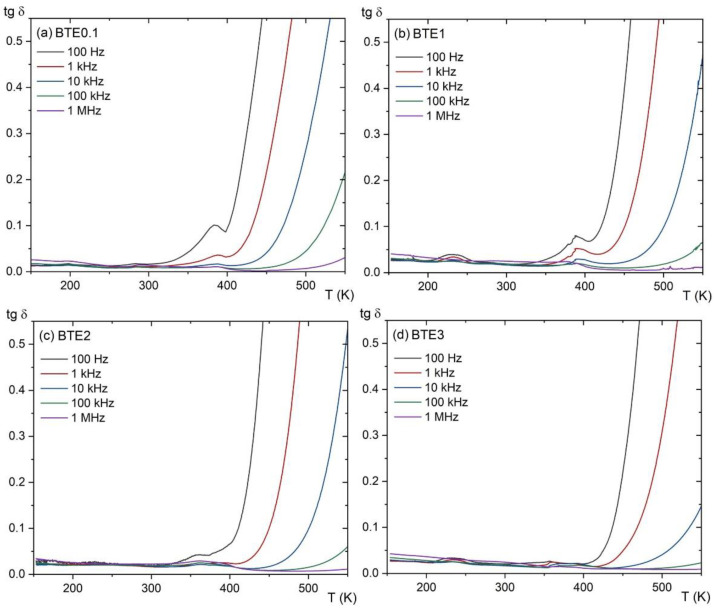
The evolution of the dielectric losses as a function of temperature at different frequencies for BTEx ceramics: (**a**) BET0.1, (**b**) BTE1, (**c**) BTE2, and (**d**) BTE3.

Figure 5 and Figure 6 display the temperature dependences of the electric permittivity (ε) and loss spectra, respectively, measured at different frequencies for the doped samples of BTEx. Similar to pure BTO, in BTE0.1 the anomaly near 400 K is associated with the tetragonal–cubic phase transition. The dielectric anomaly near 298 K is related to the orthorhombic–tetragonal (O–T) phase transition and indicates the coexistence of orthorhombic and tetragonal phases in these ceramics at room temperature. The anomaly at ~200 K is related to the lower phase transition between orthorhombic and rhombohedral phases. The incorporation of 0.1% Eu causes a slight shift in the paraelectric–ferroelectric phase transition temperature towards a lower temperature (~5 K) compared to the pure BTO.

Dielectric anomalies observed in the ε(T) dependences show typical ferroelectric behavior in all samples. The dielectric constant for pure BTO was observed with an order of 6000 at the maximum, whereas for some of the BTEx samples the dielectric constant begins to decrease as the Eu concentration increases (BTE1 is an exception). Lower values of dielectric constant usually refer to the decreasing grain size and higher porosity. The problems of grain size and porosity in relation to dielectric properties have been considered by many authors in various publications [19,20,21]. Although the BTE1 ceramic sample has a smaller grain size than BTE0.1, it has a higher sample density, resulting in lower porosity. As a result, higher values of the dielectric constant were measured. The question arises regarding whether this fact may also be connected to the imperfect stoichiometry of the samples. In the case of very small concentrations of Eu ions, they act as defects and may produce relatively large nonstoichiometry and a resulting decrease in dielectric properties. For somewhat higher concentrations, the resulting nonstoichiometry is mainly related to the oxidation state of the Eu ion, which can occur at both the 2+ and 3+ oxidation state. As a result, it can be a substitute for either Ba^2+^ or Ti^4+^ ions [22]. A similar situation was observed for BaTiO_3_ ceramics doped with Er^3+^ ions which also, depending on concentration, occupy the position of Ba or Ti in the perovskite structure of BaTiO_3_ [23]. The problem of local structural heterogeneity induced by aliovalent Eu^3+^ doping was also observed in Pb-based compounds [24]. As described in the aforementioned work, the Eu3+ doping also enchases relaxor properties of Pb-based compounds.

In our previous study [12], the detailed XPS studies showed that Eu can indeed exist at both 2+ and 3+ oxidation states and may thus occupy both A and B positions in the crystal lattice. According to [18], for concentrations of less than 3 wt.% of Eu ions, they are mainly incorporated into the A site of the perovskite structure and they act as donors. Eu^2+^ has ionic radii of 1.31 Å which is lower than the ion radii of Ba^2+^ (1.35 Å). However, according to our measurements, 2% of Eu causes the distribution of Eu^3+^: Eu^2+^ with a ratio of about 3:1. It means that, in BTE2, more Eu ions are already located in the B position than A, as Eu^3+^ [12] with 0.947 Å ionic is twice bigger than Ti^4+^ (0.42 Å). To compensate for the charge, Ti ions change their oxidation state and assume a valence of 3+ with a more comparable ionic radius (0.63 Å). From this point of view, it would appear that a concentration of about 1% gives an optimum concentration of Eu, for which the Eu^3+^/Eu^2+^ distribution has the most favorable effect on the electrical properties of the ceramics studied. With the increasing value of Eu^3+^ ions (above 1%), the ε(T) characteristics dramatically change and show the existence of a kind of plateau area before the *T*_C_. In [12], it was demonstrated that this plateau area is connected to the coexistence of ferroelectric and paraelectric phases.

### 3.4. DSC Measurements

**Figure 7:**
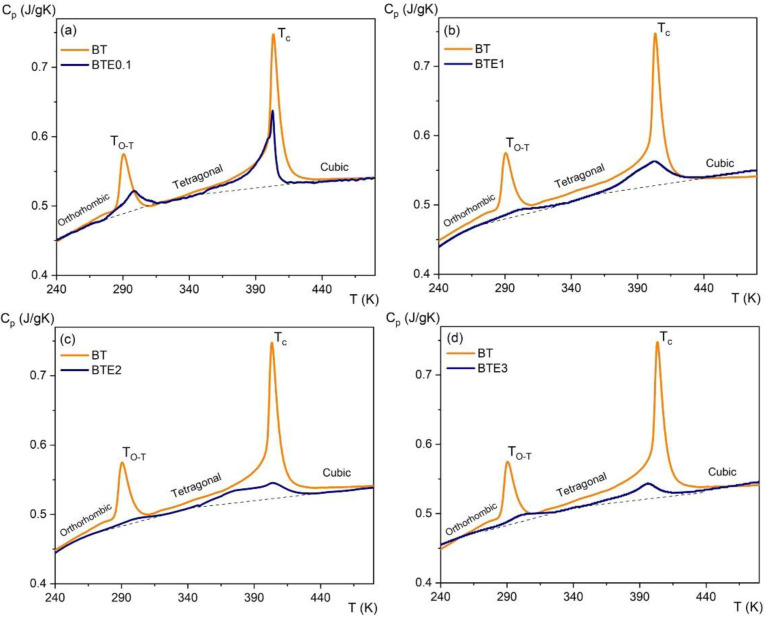
Temperature dependences of the specific heat for different compositions of BTEx ceramics: (**a**) BET0.1, (**b**) BTE1, (**c**) BTE2, and (**d**) BTE3. The dashed line illustrates the background coming from the lattice part of the specific heat.

Figure 7 presents the temperature dependences of the specific heat for different compositions of BTEx ceramics. The positions of the peaks, in the c_p_(T) dependence, correspond to structural transitions that are as follows: from orthorhombic to tetragonal symmetry at *T*_O-T_ and from tetragonal to cubic symmetry at *T*_C_. There is one more phase transition between rhombohedral and orthorhombic symmetries at low temperatures, for pure BTO at about 200 K [10] (not shown in Figure 7). For the BTO sample, the peaks are large and sharp which unambiguously defines the first-order phase transition, whereas, for BTE samples, the peaks are becoming increasingly blurred with the decreasing value of latent heat. 

A small movement of the peak positions towards lower temperatures was observed for the tetragonal–cubic phase transition as the concentration of Eu increased. The temperature of the tetragonal–orthorhombic phase transition moved slightly toward higher temperatures with Eu concentration. The temperatures of the phase transitions, and latent heat that corresponds to those transitions, are collected in Table 1.

### 3.5. Raman Scattering

**Figure 8:**
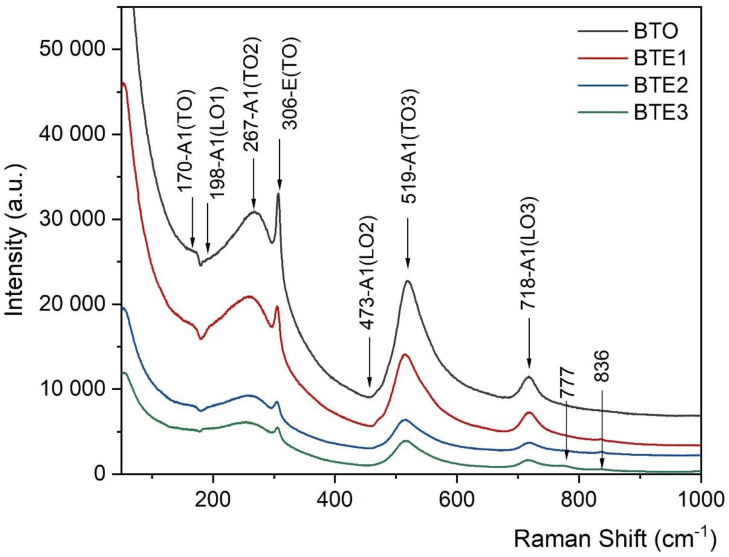
Room temperature Raman spectra of the pure BTO and for BTEx (BTE1, BTE2, BTE3) ceramics.

BTO exhibits a tetragonal symmetry belonging to the space group *P4mm*. For this symmetry, the selection rules predict 13th first-order Raman modes between 100 cm^−1^ and 900 cm^−1^ [25]. However, in ceramics, due to random grain orientation, the unknown scattering geometries make mode assignment based on group theory less unambiguous [25]. For this purpose, only the dominant modes can be distinguished within peak convolutions, as noted in Figure 8 (for BTO). Figure 8 also gives the room temperature Raman spectra for doped samples of BTEx. Two distinct bands can be distinguished from the spectrum at 170 and 306 cm^−1^ and three asymmetric broader bands at 270, 520, and 720 cm^−1^. The latter three bands are still present in the cubic para-electric phase but are much broader and more symmetrical. They have often been attributed to second-order effects in the literature but are more likely to be related to disordered Ti displacements in octahedra. The extremely broad and weak band at 720 cm^−1^ in the paraelectric phase is considered as a precursor of the tetragonal phase. BTO exhibits a tetragonal symmetry belonging to the space group C4v. Similar spectra for the tetragonal phase have been well described in the literature [26]. As indicated in the literature, the peak observed at 305 cm^−1^ corresponds to the E(TO2) phonon mode of tetragonal BTO. The modes that were observed at about 170, 267, 519, and 718 cm^−1^ are designated as A1(TO1), A1(TO2), A1(TO3), and A1(LO3), respectively. Raman spectra obtained from Eu^3+^-doped BT did not show any unusual wavelength shift. The only two new modes are located near 777 and 836 cm^−1^ and can be observed for BTE3.

All Raman modes also become noticeably weaker and broader with an increase in Eu concentration. The broadening of the Raman modes results from structural disorder that appears due to substitution with ions of different valence states and/or ionic radii. It also indicates that the higher Eu concentration results in worse crystallinity, which is in agreement with already presented SEM results. For higher doping (BTE3), new modes appear, as indicated in Figure 8 by arrows. These new/extra modes appear due to localized phonon vibrations in the vicinity of a (substitutional) defect or as a result of broken selection rules due to defect-induced disorder [27].

### 3.6. Ferroelectric Measurements

**Figure 9:**
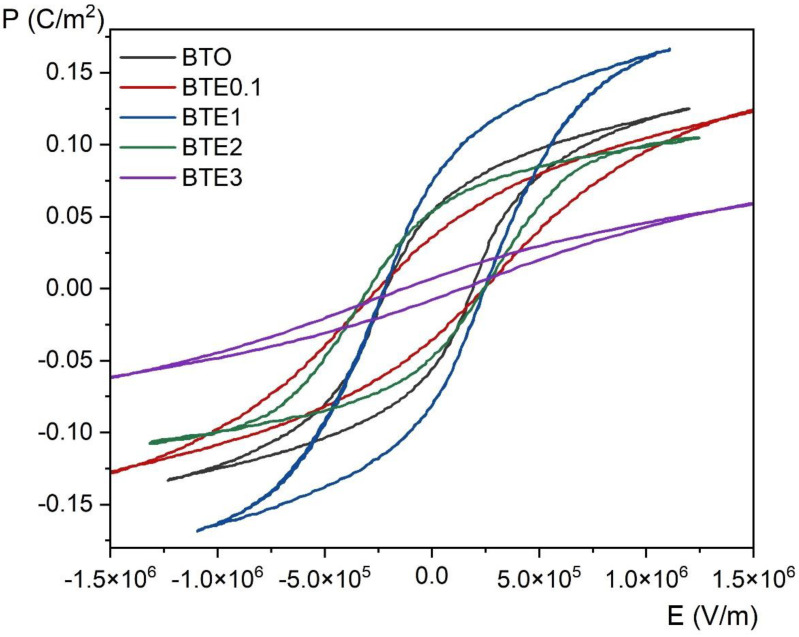
Room temperature *P*(*E*) hysteresis loops for pure BTO and BTEx (BTE0.1, BTE1, BTE2, BTE3) ceramics.

In Figure 9, P-E hysteresis loops for all BTEx samples measured at room temperature are shown, including pure BTO (for comparison purposes). The saturated *P*(*E*) loops indicate pure ferroelectric specimen characteristics. The effect of the degree of doping with Eu ions on the remnant polarization (*P*r) and the coercive field (*E*c) can be clearly discerned. All presented *P*(*E*) loops manifest typical ferroelectric behavior. In the case of pure BTO, the values of *P*r (~2 × 10^5^ V/m) and the coercive field are similar to those presented in the literature for ceramic samples [28]. It was already mentioned that grain size considerably influences ceramics’ dielectric and electric properties. According to the statement presented in [28], the increase in grain size results in easier rotation of the domain walls due to an increase in domain switching, which improves ferroelectric properties. Thus, it should not be surprising that as the concentration increases, the remaining polarization decreases due to lowering grain sizes. The exception is BTE1, where the polarization visibly increases. The increasing ferroelectric properties for BTE1 may be the result of the above mentioned most favorable ratio of Eu^3+^/Eu^2+^—just 1% concentration of Eu ions—as well as the high density of the BTE1 sample, as described in Section 3.2.

### 3.7. Electrocaloric Properties

**Figure 10:**
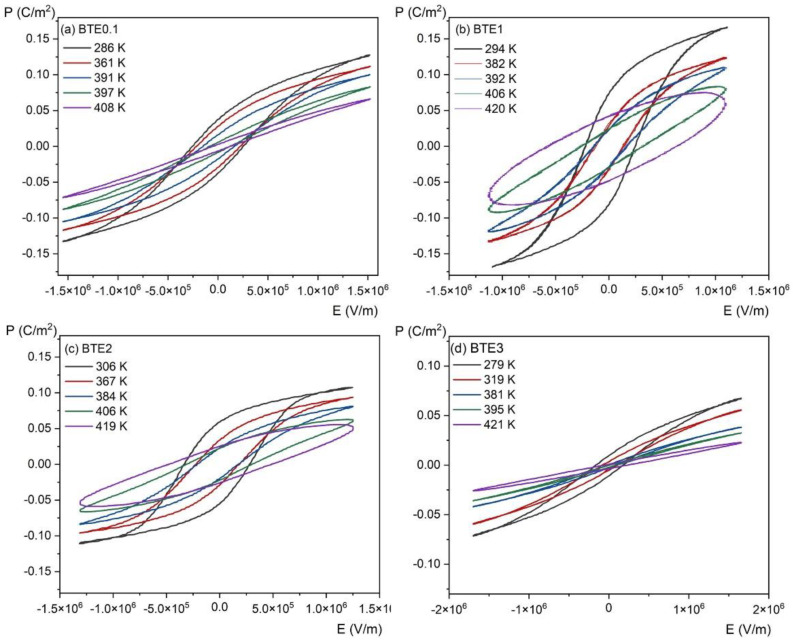
Temperature-dependent *P*(*E*) hysteresis loops of BTEx ceramics: (**a**) BET0.1, (**b**) BTE1, (**c**) BTE2, and (**d**) BTE3.

**Figure 11:**
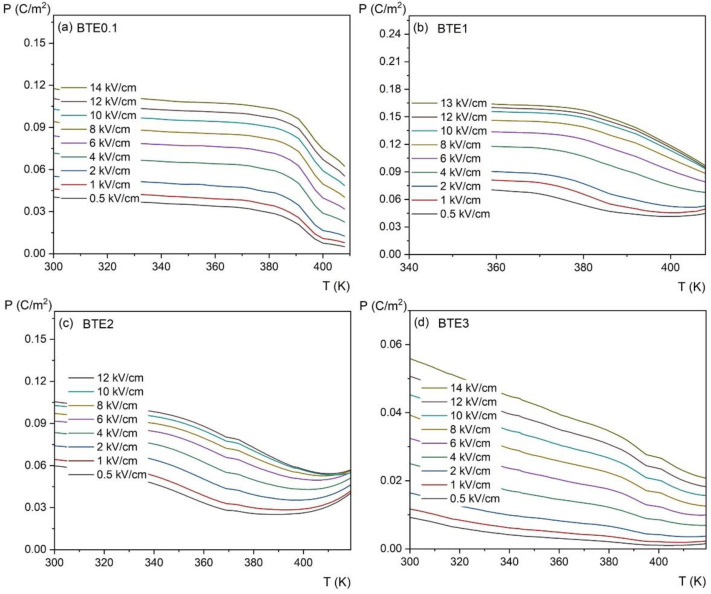
Temperature dependence of the spontaneous polarization measured for BTEx ceramics: (**a**) BET0.1, (**b**) BTE1, (**c**) BTE2, and (**d**) BTE3 measured at different electric fields.

To estimate the electrocaloric effect (ECE) by indirect measurement, *P*(*E*) hysteresis loops were investigated as a function of temperature. Figure 10 shows the variation of *P*(*E*) loops for different temperatures, while Figure 11 shows the temperature dependence of polarization *P*(*T*) for the same set of compositions. Polarization gradually decreases with increasing temperature due to thermal vibrations of the crystal lattice, which gradually destroys the ordering of dipoles and leads to a phase transition.

Usually, the intensity of the ECE is qualitatively denoted by the temperature change Δ*T* in the adiabatic process [29]:

(1) $$\Delta T=-\frac{1}{C_{p}\rho }\int_{E_{1}}^{E_{2}}T{\left(\frac{\partial P}{\partial T}\right)}_{E}dE$$

where *ρ* is the density of the samples used in measurements, *C_p_* is the specific heat at a given temperature (taken from the measurements shown in Figure 5), ${\left(\frac{\partial P}{\partial T}\right)}_{E}$ represents the variation rate of polarization versus temperature under a constant electric field, and *E*_1_ and *E*_2_ are the initial and final applied electric fields (normally *E*_1_ = 0 and *E*_2_ = *E*). Coefficient $\frac{\partial P}{\partial T}$ is obtained by numerical differentiation of the *P*(*T*) data presented in Figure 11 which were derived from the upper branches of the P-E loops presented in Figure 10 and for *E* > 0.

**Figure 12:**
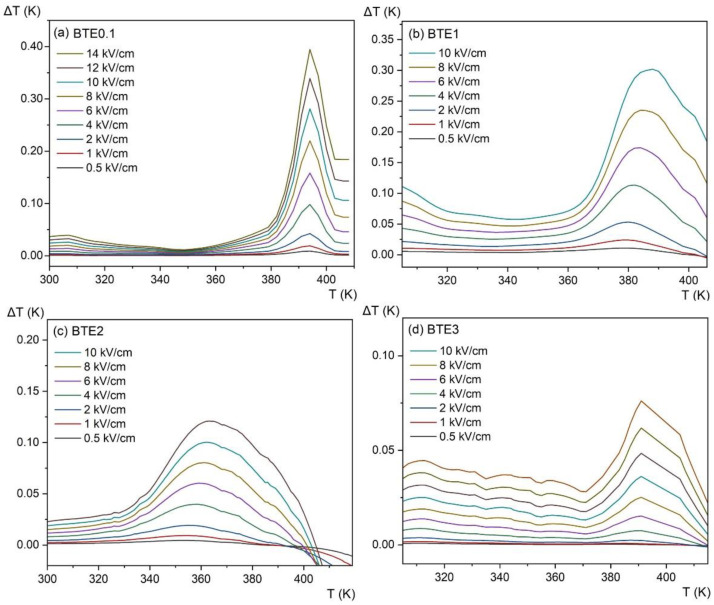
Electrocaloric temperature change (ΔT) as a function of temperature at different applied fields for BTEx ceramics: (**a**) BET0.1, (**b**) BTE1, (**c**) BTE2, and (**d**) BTE3.

Figure 12 shows the adiabatic change in electrocaloric temperature (Δ*T*) for a few electric fields and for all compositions. The observed anomalies (maxima) are due to phase transitions that are greatly enhanced by the applied electric field. Compared to pure BTO, where the EC effect reached the value Δ*T* = 0.6 K [14], the value of ECE for doped samples decreases along with the increase in Eu ion concentration. This lowering EC effect is mainly related to decreasing polarization which decreases due to lowering grain size as well as the stoichiometry problems described in Section 3.3. However, it is worth mentioning that the heavily diffused peak of the temperature change of ECE (Δ*T*) observed for the BTE3 sample gives a larger temperature window, which can be beneficial for micro-refrigeration applications. While the value of Δ*T* decreases with an increasing amount of Eu, the negative value of the electrocaloric effect above 400 K visible in BTE2 is also an interesting feature. This effect is most probably related to induced polarization coming from polar micro and/or nano-regions that are present in the paraelectric phase [12,14,30]. The presence of space charge inducing electrical conductivity also has an impact here, assuming that the electrocaloric effect values obtained for the ceramics of BTE are relatively small. However, taking into account that the largest ECE effect for bulk materials belongs to lead-based Pb_0.99_Nb_0.02_(Zr_0.75_Sn_0.20_Ti_0.05_)_0.98_O_3_ material [31,32] which achieves the value of 2.5 K around *T*c, we believe that the search for lead-free compositions with improving values of ECE, such as those based on ferroelectric barium titanate, is worth continuing.

## 4. Conclusions

The effect of Eu doping on lead-free BaTiO_3_ ceramics was examined. The samples were successfully synthesized by the conventional method. Both X-ray diffraction and Raman spectroscopy showed tetragonal symmetry for all doped samples at room temperature. For higher concentrations (BTE3), a weak influence of the orthorhombic phase can be visible. The appearance of additional Raman modes in higher-doped BTE is directly related to the existence of local disorder in the crystal lattice as well as the presence of defects. A slight decrease in Curie temperature (*T*_C_) due to doping has been revealed, while at the same time the *T*_O-T_ temperature increases. Ferroelectric properties appeared to be the finest for BTE1 ceramic. With an increasing amount of Eu (especially for BTE3), a significant decrease in spontaneous polarization value (*P*s from ~0.15 to ~0.07 C/m^2^) can be observed which may be mostly related to the worse crystalline, i.e., smaller grain size, higher porosity, and consequently lower density of the samples.

## Figures and Tables

**Table 1:** Temperatures of dielectric anomalies corresponding to successive phase transitions in BTEx ceramics together with the values of latent heat Δ*E* corresponding to a particular phase transition.

Sample	*T*_C_ (K)	Δ*E* (J/g)	*T*_O-T_ (K)	Δ*E* (J/g)
BTO	404	2.797	290	1.14
BTE0.1	403	1.534	298	0.75
BTE1	402	1.454	299	0.342
BTE2	401	1.174	301	0.132
BTE3	395	0.539	302	0.269

## Data Availability

The data presented in this study are available on request from the corresponding author.
